# Evaluation of Ultrasound Elastography Combined With Chi-Square Automatic Interactive Detector in Reducing Unnecessary Fine-Needle Aspiration on TIRADS 4 Thyroid Nodules

**DOI:** 10.3389/fonc.2022.823411

**Published:** 2022-02-16

**Authors:** Xiao Liu, Li Xie, Xianjun Ye, Yayun Cui, Nianan He, Lei Hu

**Affiliations:** Department of Ultrasound, The First Affiliated Hospital of University of Science and Technology of China (USTC), Division of Life Sciences and Medicine, University of Science and Technology of China, Hefei, China

**Keywords:** decision tree, shear wave elastography, stiffness, thyroid nodule, ultrasound

## Abstract

**Background:**

Conventional ultrasound diagnosis of thyroid nodules (TNs) had a high false-positive rate, resulting in many unnecessary fine-needle aspirations (FNAs).

**Objective:**

This study aimed to establish a simple algorithm to reduce unnecessary FNA on TIRADS 4 TNs using different quantitative parameters of ultrasonic elasticity and chi-square automatic interactive detector (CHAID) method.

**Methods:**

From January 2020 to May 2021, 432 TNs were included in the study, which were confirmed by FNA or surgical pathology. Each TN was examined using conventional ultrasound, sound touch elastography, and Shell measurement function. The quantitative parameters *E* and *E*_shell_ were recorded, and the *E*_shell_/*E* values were calculated for each TN. The diagnostic performance of the quantitative parameters was evaluated using the receiver operating characteristic curves. The CHAID was used to classify and analyze the quantitative parameters, and the prediction model was established.

**Results:**

A total of 226 TNs were malignant and 206 were benign. *E*_shell_ and *E*_shell_/*E* ratio were included in the classification algorithm, which showed a depth of two ramifications (*E*_shell_/*E* ≤ 0.988 or 0.988–1.043 or >1.043; if *E*_shell_/*E* ≤ 0.988, then *E*_shell_ ≤ 64.0 or 64.0–74.0 or >74.0; if *E*_shell_/*E* = 0.988–1.043, then *E*_shell_ ≤ 66.0 or > 66.0; if *E*_shell_/*E* >1.043, then *E*_shell_ ≤ 69.0 or >69.0). The unnecessary FNAs could have been avoided in 57.3% of the cases using this algorithm.

**Conclusion:**

The prediction model using quantitative parameters had high diagnostic performance; it could quickly distinguish benign lesions and avoid subjective influence to some extent.

## Introduction

Thyroid nodules (TNs) have become a prevalent clinical disease. The detection rate of TNs has gradually increased in China, as the mass population has begun to pay attention to physical examination every year and ultrasound technology is constantly updated. Thyroid cancer is a common head and neck malignant tumor (1–3), which has become a hot topic among doctors and patients.

Ultrasound is the preferred method for thyroid examination in clinical practice to assess TNs and cervical lymph nodes (4, 5). In recent years, many TN risk stratification systems (RSSs) have been introduced to express the descriptions of TNs by radiologists relatively objectively, which are based on suspected US features (solid, very hypoechoic, taller-than-wide, extra-thyroidal extension, punctate echogenic foci, and so on) of a TN (6–8). In 2017, the American College of Radiology (ACR) published a white paper on the classification and diagnosis of TNs. The ACR-TIRADS was used to assign points for the suspected US features of a TN. The total points determined the ACR-TIRADS level from TIRADS 1 to TIRADS 5. When the maximum diameter of TIRADS 4 nodule was greater than or equal to 15 mm or a TIRADS 5 nodule was greater than or equal to 10 mm, the fine-needle aspiration (FNA) was needed. The ACR-TIRADS was developed to maximize the identification of malignant tumors using a unified approach, avoiding unnecessary FNA, while not losing cancers avoided among FNAs. Among these, the malignant risk of ACR-TIRADS 4 of TNs was about 5%–20%. This probability span was large, and the false-positive rate was high. In practice, doctors sometimes recommend FNA for patients with TIRADS 4 of TNs because of their anxiety and fear, but the puncture and follow-up of some patients show benign results, which increases the unnecessary burden.

ACR-TIRADS does not include the characteristics of ultrasound elastography (UE). At present, many studies have confirmed the excellent diagnostic performance of UE in thyroid diseases (9–11). Some studies showed that TNs could be differentiated by measuring the internal stiffness of nodules with a sensitivity of 77.2%–85.7% and a specificity of 80.5%–96.0%. The stiffness of benign TNs was lower than that of malignant TNs (12–14).

However, hemorrhage, calcification, and cystic changes may occur in TNs, leading to uneven internal echo, which affects the measurement of TN stiffness using UE. In actual measurements, the internal stiffness of TNs can be quantitatively assessed by sound touch elastography (STE) and peripheral stiffness using the Shell measurement function. Our previous study confirmed that measuring a tissue stiffness of 2 mm around TNs using the Shell measurement function could improve the accuracy of differentiating between malignant and benign TNs (15, 16).

Classification algorithms aim to aid in clinical decision making by incorporating different criteria in a formalized manner. For example, an official and impersonal combination of diagnostic features in the context of a multivariable approach is supposed to improve specificity and reduce variability (17). CHAID is a commonly used decision tree algorithm. Its purpose is to divide the total research population into several relatively homogeneous subpopulations based on certain characteristics (independent variable values) for analysis. Some variables are selected by the decision tree from all independent variables for analysis according to their contribution. Hence, the decision tree can automatically process a large number of independent variables and is more adaptable. CHAID is a nonparametric algorithm; therefore, it is not limited by too many applicable conditions. It has a wider application range and is more suitable for analyzing various complex connections, especially for the analysis of samples with nonlinear correlation or interaction. Hence, it is better than the ordinary statistics model. Some studies applied this algorithm to magnetic resonance imaging and obtained positive results (18–20). Only a few studies applied this algorithm to breast ultrasound research (21). This classification algorithm has not been established for thyroid research yet.

Therefore, this novel study aimed to classify and analyze ultrasound elasticity parameters, select appropriate nudes, combine different parameters, and build a predictive model to objectively assist in the clinical management of TNs and reduce unnecessary FNA on TIRADS 4 nodules.

## Materials and Methods

### Patient Selection

The ethics committee of the First Affiliated Hospital of the University of Science and Technology of China (USTC) approved this prospective study, and all patients gave informed consent to be included in the study. From January 2020 to May 2021, 1179 consecutive patients with 1463 TNs in the First Affiliated Hospital of USTC were detected by conventional ultrasound. All the TNs were classified according to ACR-TIRADS (7), and then TIRADS 4 TNs were selected. Next, we continued to select the following nodules for this study: solid or <20% cystic; at least 2-mm thyroid tissue around TNs could be measured. All TNs were confirmed by final surgical pathology or FNA.

Ultrasound-guided FNA on TNs was performed by two radiologists having more than 3 years of experience in FNA. According to the Bethesda System for Reporting Thyroid Cytopathology (22), the cytological reports were issued by one of three pathologists with more than 5 years of experience in diagnosing thyroid cytological pathology. All benign or malignant TNs included in the study were confirmed by FNA reports (Bethesda II, V, and VI) or definite histopathological reports. If the FNA report was Bethesda I, another FNA was performed on the same nodule 2 weeks later. TNs whose FNA was reported as Bethesda III or IV and that had no subsequent defined postoperative pathology were excluded. TNs considered benign were included as follows: FNA reports were Bethesda II, and they were followed up for 6 months with the maximum diameter increased by less than 20% and less than 2 mm, or the volume increased by less than 50% on conventional ultrasound (7, 23). All the TNs considered malignant by FNA were further confirmed by histopathology.

**Figure 1:**
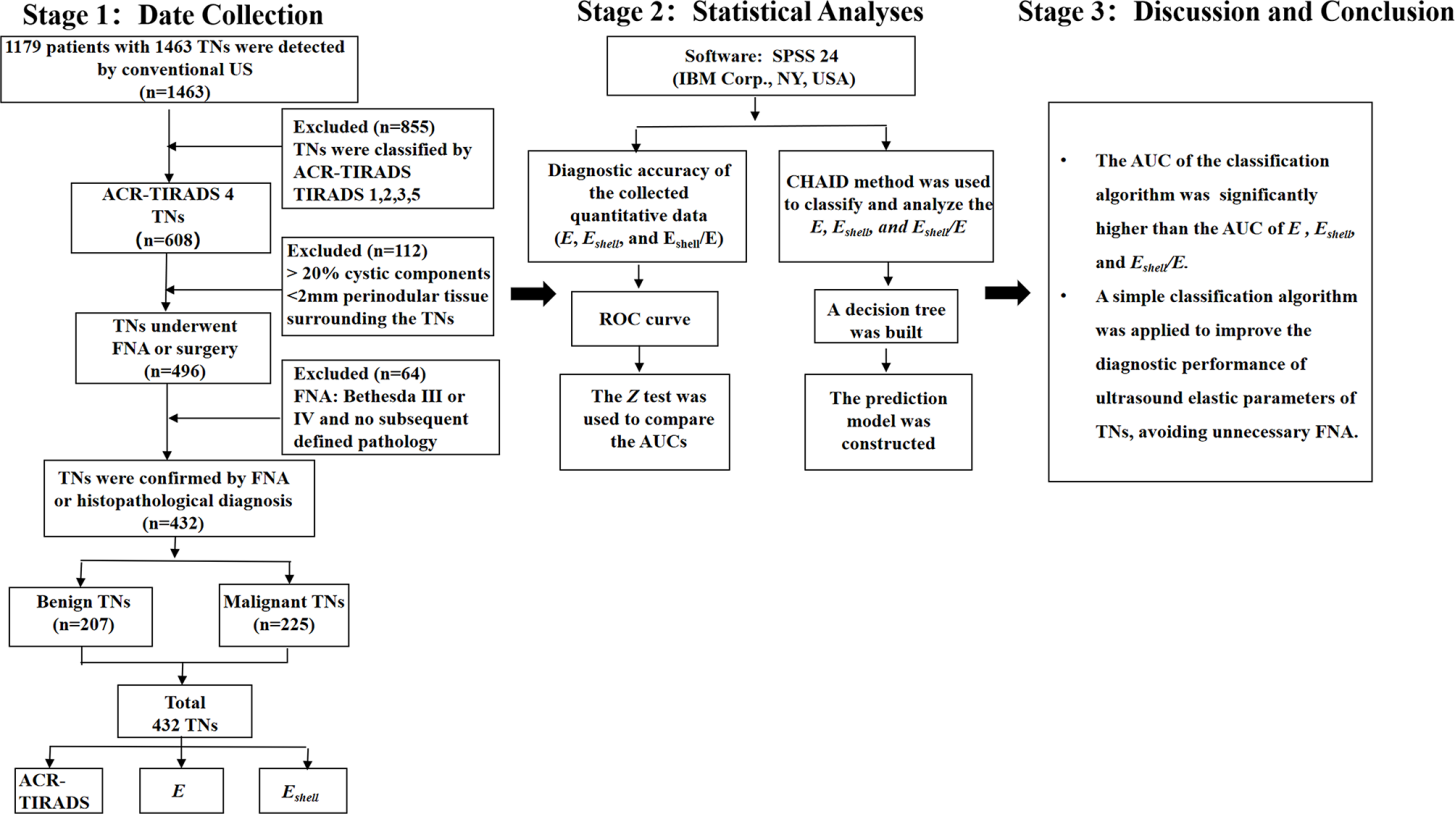
Flowchart of the methodology.

Among multiple TNs in the ipsilateral lobe of the thyroid, the ones with the highest risk of malignancy were selected. If the risk was similar, the largest one was selected. Eventually, 364 patients with 432 TNs were included in the study (**Figure 1**).

### Ultrasound Instrument

**Figure 2:**
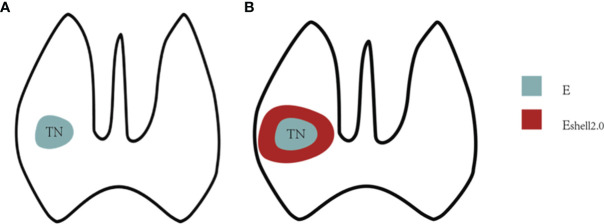
Shell measurement diagram. **(A)** *E* value was the interior stiffness of the TN. **(B)** *E*_shell_ value referred to 2 mm-perinodal stiffness of the TN.

The ultrasound diagnostic instruments used in this study were a Resona 7 US diagnostic system (Mindray Medical Solutions, Shenzhen, China) and an 11L3 transducer, with the STE function and the Shell measurement software. The Shell software automatically measured the stiffness of the tissue around the target nodules, with a range of 0.5–9 mm and increments of 0.5 mm (**Figure 2**).

### Conventional Ultrasound

The ACR-TIRADS classification of all conventional ultrasound images was performed by two radiologists with 10 and 12 years of experience in ultrasound diagnosis, and a consensus was reached after discussion in the case of any disagreement. Neither radiologist had any knowledge of the clinical data of any of the patients.

### UE Image Acquisition

**Figure 3:**
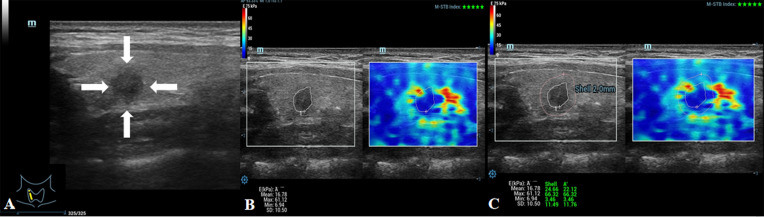
Images showing an ACR-TIRADS 4 TN in a 48-year-old female patient. The pathological diagnosis after surgery was papillary thyroid carcinoma. **(A)** Conventional ultrasound image of the TN; the arrows point to the TN. **(B)** *E* value of the TN was 61.12 kPa. **(C)** *E*_shell_ value of the TN was 66.32 kPa.

**Figure 4:**
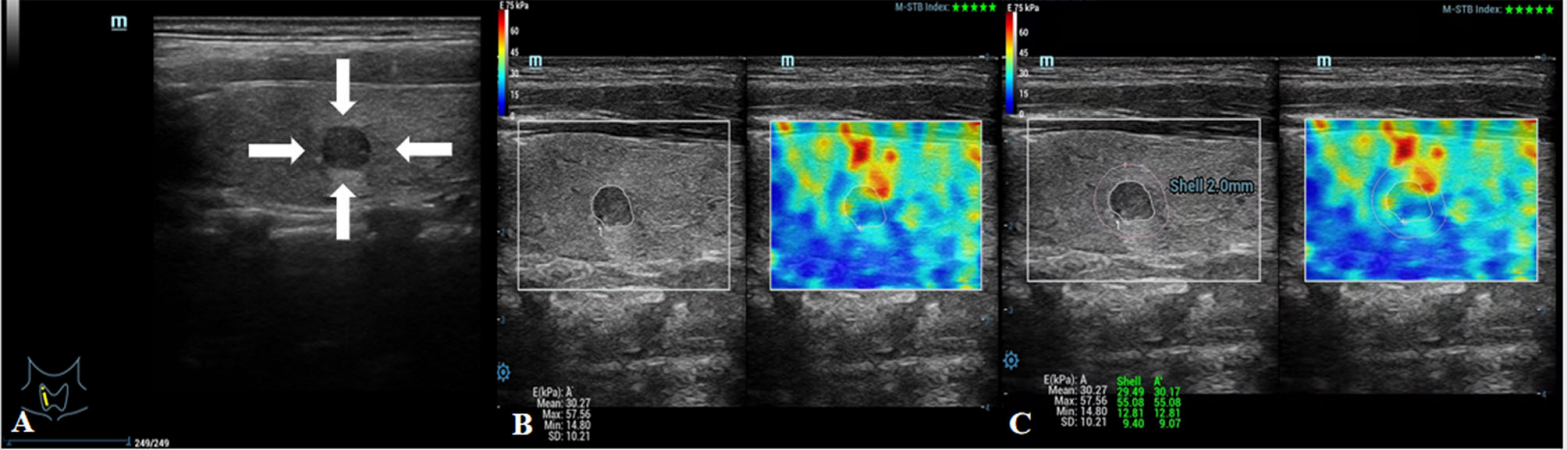
Images showing an ACR-TIRADS 4 TN in a 24-year-old female patient. The FNA diagnosis was a benign TN. **(A)** Conventional US image of the TN; the arrows point to the TN. **(B)** *E* value of the TN was 57.56 kPa. **(C)** *E*_shell_ value of the TN was 55.08 kPa.

First, the longitudinal section of the lateral lobe where the TN was located was selected. The TN was then put in the middle of the region of interest (ROI) to ensure that the ROI included the TN and at least 2 mm of the surrounding thyroid tissue, and the instrument was adjusted to the best state. Then, the edges of the TN were delineated using a tracing method (STE examination), and the internal stiffness of the nodules was measured as the *E* value. Next, the Shell function was activated, and the stiffness of 2-mm tissue around the nodule was selected to be measured as the *E*_shell_ value (**Figures 3**, **4**). The stiffness of each nodule and its periphery was measured three times and averaged. A ratio of the *E*_shell_/*E* was calculated using the acquired *E* and *E*_shell_ values. The patients were asked to hold their breath so as to reduce the impact of breathing on measurements. All patients were attended by the same radiologist, who had 10 years of experience in ultrasound diagnosis and 5 years in thyroid UE.

### Statistical Analysis

The software SPSS 24 (IBM Corp., NY, USA) was used for statistical analysis. Quantitative data were shown as the mean ± SD. Qualitative data were shown as frequencies. The *χ* ^2^ test and Fisher’s exact probability test were used to compare categorical variables. First, the diagnostic accuracy of the collected quantitative data (*E*, *E*_shell_, and *E*_shell_/*E*) was evaluated using the receiver operating characteristic (ROC) curve. Then, the exhaustive chi-squared automatic interaction detection (CHAID) method was used to classify and analyze the elastic data. Based on the chi-square test results, a decision tree was built using the CHAID method. Minimal parent and child node sizes were set to 10 and 5, respectively. The robustness of the classification tree was verified by tenfold cross-validation. The CHAID algorithm automatically calculated the cutoff values of each ramification, and the prediction model was constructed. Therefore, predefined cutoff values were not used. The differences in quantitative parameters were detected with the independent-sample *t* test. The *Z-*test was used to compare the areas under ROC curve. A *P* value < 0.05 was considered to indicate a statistically significant difference.

## Results

### Conventional Data and US Features

The characteristics of participants are shown in **Table 1**. The study included 432 TNs in 398 patients; of these, 364 had one single nodule and 34 had one nodule in each lobe. The median age was 48 years (range, 15–79 years); 76.7% (305/398) were women. The size of malignant TNs (maximum diameters were measured using grayscale ultrasound) was significantly smaller than the size of benign TNs (9.73 ± 2.31 vs 12.51 ± 2.41 mm, *P* < 0.01).

**Table 1:** Characteristics of participants.

Characteristics	Total	Malignant	Benign
Patients, *n*	398	201	197
Age, year	46.40 ± 12.05	43.41 ± 10.54	49.45 ± 12.73
Sex (male, female)	93/305	58/143	35/162
One single TN, *n*	364	176	188
Two TNs, *n*	34	25	9
TNs, *n*	432	226	206
Size of TNs, cm	11.05 ± 2.74	9.73 ± 2.31	12.51 ± 2.41

### Pathological Diagnosis

A total of 226 TNs were malignant (52.3%) and 206 were benign (47.7%). All the benign TNs had the diagnosis of Bethesda category II and were followed up for 6 months. All the malignant TNs had the diagnosis of Bethesda category IV or V, and every nodule was confirmed by postoperative pathology, which were all papillary thyroid carcinomas.

### *E* and *E*_shell_ Values

In malignant TNs, the *E*_shell_ values were higher than the *E* values, and the difference was statistically significant (*P* < 0.01). In benign TNs, the *E*_shell_ values were lower than the *E* values, and the difference was statistically significant (*P* < 0.01). Compared with benign TNs, the *E* values, *E*_shell_ values, and *E*_shell_/*E* values of malignant TNs were higher, and the difference was statistically significant (all *P* < 0.01) (**Table 2**).

**Table 2:** Average *E*, *E_shell_* and *E_shell_/E* ratio of malignant and benign TNs.

	malignant	benign	*P* (malignant vs. benign)
*E* (kPa)	67.54 ± 12.45	61.50 ± 10.34	0.000*
*E_shell_* (kPa)	76.49 ± 13.55	58.70 ± 10.24	0.000*
*E_shell_/E*	1.144 ± 0.145	0.962 ± 0.134	0.000*
*P* (*E* vs. *E_shell_*)	0.000*	0.006*	

*P -values listed are less than 0.05.

### Diagnostic Performance of Quantitative Parameters

**Figure 5:**
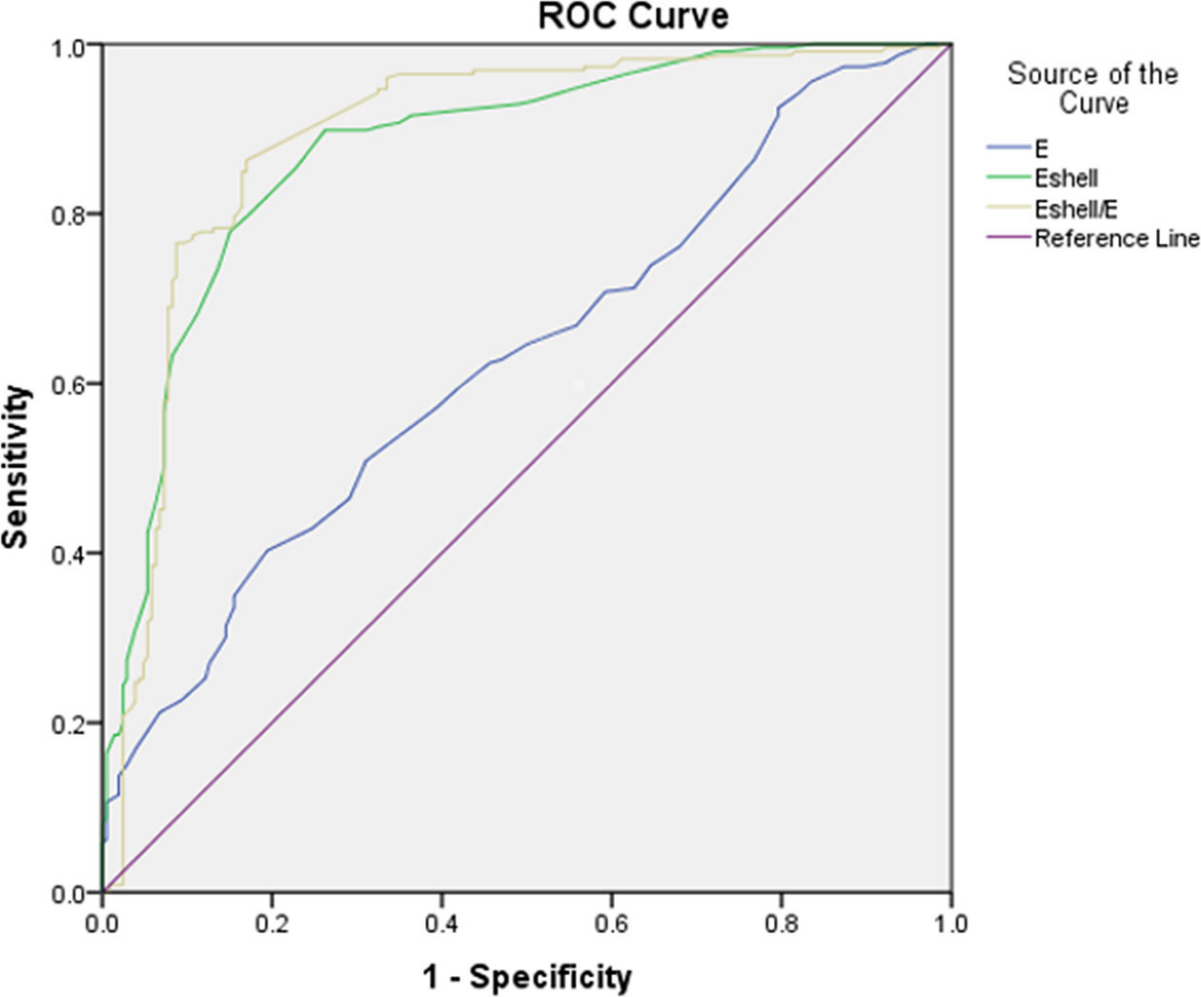
Receiver operating characteristic curves of *E*, *E*_shell_, and *E*_shell_/*E* to analyze diagnostic performance (AUC of *E* = 0.629; AUC of *E*_shell_ = 0.876; AUC of *E*_shell_/*E* = 0.890).

In all TIRADS 4 TNs, *E*_shell_ values showed the highest sensitivity (89.8%, *P* < 0.05), while the *E*_shell_/*E* values had significantly higher specificity (83.0%, *P* < 0.05) in differentiating malignant from benign TNs (**Table 3**). In the ROC curve analysis, the three parameters, the *E*_shell_, *E*, and *E*_shell_/*E* values, showed good diagnostic performance [*E* values: area under the curve (AUC) 0.629, cutoff 69.50, *P* < 0.05; *E*_shell_ values: AUC 0.876, cutoff 64.50, *P* < 0.05; *E*_shell_/*E* ratio: AUC 0.890, cutoff 1.005, *P* < 0.05] (**Figure 5**).

**Table 3:** Diagnostic performance of all acquired quantitative parameters.

	Sensitivity	Specificity	Cutoff	95%CI	AUC	*P*
*E*	40.3	80.6	69.50	57.7-68.1	0.629	0.000
*E_shell_*	89.8	73.8	64.50	84.3-91.0	0.876	0.000
*E_shell_/E*	86.3	83.0	1.005	85.7-92.4	0.890	0.000

Cutoff values of E, E_shell_ are given in kPa. Sensitivity, specificity, and 95% confidence intervals (CI) in %.

### Classification Algorithm

**Figure 6:**
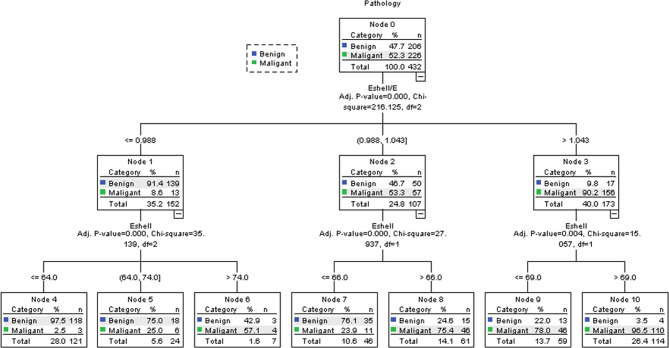
Decision tree of the CHAID. Using the parameters with the highest discriminating power (*E*_shell_ and *E*_shell_/*E*), the research population (Node 0) was further split into child nodes (1–10). After two ramifications, no further discrimination could be achieved. Green tags indicate the percentage of malignant TNs, while blue tags indicate the percentage of benign TNs in each node.

The results of the classification algorithm are shown in **Figure 6** and **Table 4**. The results included the *E*_shell_ and *E*_shell_ ratio values, but the *E* values were not included in the algorithm because they did not improve the accuracy of the algorithm. First, the *E*_shell_/*E* ratio was evaluated: if the *E*_shell_ values were ≤0.988, the probability of malignancy was 8.6% (Node 1) and the *E*_shell_ values were considered; when its values were ≤64.0, the probability of malignancy dropped to 2.5% (Node 4); when its values were = 64.0–74.0, the probability of malignancy was 25.0% (Node 5); when its values were > 74.0, the probability of malignancy was 57.1% (Node 6). If the *E*_shell_ values were 0.988–1.043, the probability of malignancy was 53.3% (Node 2) and the *E*_shell_ value was considered; when its values were ≤ 66.0, the probability of malignancy was 23.9% (Node 7); and when its values were ≤66.0, the probability of malignancy was 75.4% (Node 8). If the *E*_shell_/*E* ratio >1.043, the probability of malignancy was 90.2% (Node 3) and the *E*_shell_ values were considered; when its values were ≤ 69.0, the probability of malignancy was 78.0% (Node 9); and when its values were > 69.0, the probability of malignancy was 96.5% (Node 10).

**Table 4:** Characteristics of the nodes of the classification algorithm.

Node	Definition	Predicted category	malignant n (%)	benign n (%)	total n
1	*E_shell_*/*E* ≤0.988	benign	13 (8.6)	139 (91.4)	152
2	*E_shell_*/*E* 0.988-1.043	malignant	57 (53.3)	50 (46.7)	107
3	*E_shell_*/*E*>1.043	malignant	156 (90.2)	17 (9.8)	173
*4*	*E_shell_/E ≤0.988 and E_shell ≤_64.0*	*benign*	*3 (2.5)*	*118 (97.5)*	*121*
*5*	*E_shell_/E ≤0.988 and E_shell_ 64.0-74.0*	*benign*	*6 (25.0)*	*18 (75.0)*	*24*
*6*	*E_shell_/E ≤0.988 and E_shell_＞74.0*	*malignant*	*4 (57.1)*	*3 (42.9)*	*7*
*7*	*E_shell_/E 0.988-1.043 and E_shell ≤_66.0*	*benign*	*11 (23.9)*	*35 (76.1)*	*46*
*8*	*E_shell_/E 0.988-1.043 and E_shell_＞66.0*	*malignant*	*46 (75.4)*	*15 (24.6)*	*61*
*9*	*E_shell_/E＞1.043 and E_shell ≤_69.0*	*malignant*	*46 (78.0)*	*13 (22.0)*	*59*
*10*	*E_shell_/E＞1.043 and E_shell_＞69.0*	*malignant*	*110 (96.5)*	*4 (3.5)*	*114*

E_shell_ are given in kPa.

The table refers to the nodes in **Figure 6**. Nodes 1,2 and 3 represent parent nodes, and nodes 4-10 (italics) represent terminal nodes.

The AUC of the classification algorithm was 0.926 (95% CI 0.901–0.951, *P* < 0.01), which was significantly higher than the AUC of *E* (0.629, *Z* = 10.10, *P* < 0.01), *E*_shell_ (0.0.876, *Z* = 2.38, *P* < 0.01), and *E*_shell_/*E* (0.890, *Z* = 1.68, *P* < 0.01). The sensitivity of the classification algorithm was 98.7% (223/226), and the specificity was 57.3% (118/206) (**Table 5**).

**Table 5:** Comparison of classification algorithm and pathological results.

Classification algorithm	Pathological results		total
	malignant	benign	
malignant	223	88	311
benign	3	118	121
total	226	206	432

Using this algorithm, 118 out of 206 benign TNs were correctly classified. Hence, the unnecessary FNA could have been avoided in 57.3% of the cases. The algorithm produced three false-negative cases (2.5%).

## Discussion

The results of this study showed that a simple prediction model was established by combining the two quantitative indexes (*E*_shell_ and *E*_shell_/*E*) with the classification algorithm, which could accurately distinguish the benign and malignant TNs and reduce the FNA rate of the TIRADS 4 nodules.

At present, several versions of RSSs exist worldwide. Some studies confirmed that ACR-TIRADS had high diagnostic efficiency in differentiating between benign and malignant TNs and a better performance in avoiding unnecessary FNA, but did not have the highest sensitivity among RSSs (16, 24–26). For TIRADS 4 TNs, many FNA results were benign, which increased the unnecessary puncture risk and economic burden for patients (6, 26).

As a relatively new ultrasound technology, the STE had the advantage that it could automatically measure the stiffness of the target using the software to get the quantitative values, and hence the evaluation was relatively objective. A large number of studies confirmed the value of STE in the diagnosis of TNs (27–29).

Most malignant TNs attracted the attention of radiologists when their diameters were small and necessary intervention measures could be taken. Therefore, in our study, the maximum diameters of malignant TNs were significantly smaller than those of benign TNs (*P* < 0.01). Although the maximum diameter of malignant TNs was smaller than that of benign TNs, their internal stiffness (*E*) was significantly higher than that of benign TNs (*P* < 0.01, **Table 2**).

However, the measurement of internal stiffness of nodules could be subject to errors because the internal stiffness of nodules was often affected by factors such as calcification. The stiffness around the nodules was not affected by these factors. This study confirmed that the internal (*E*) and peripheral stiffnesses (*E*_shell_) of malignant TNs were higher than those of benign nodules (both *P* < 0.01). The peripheral stiffness of malignant TNs was higher than their internal stiffness (*P* < 0.01), which was due to the increase in collagen fiber composition around malignant TNs. In particular, the 2-mm area around the nodules was more abundant in collagen fibers, which increased the peripheral stiffness of malignant TNs. Because of this pathological mechanism, it was meaningful to measure the stiffness around nodules using the STE, which was not affected by the uneven echo inside nodules and hence was more objective (15, 29). On the contrary, benign nodules did not stimulate the proliferation of peripheral fibroblasts, and therefore the peripheral stiffness did not increase. In this study, the peripheral stiffness of benign TNs was lower than the internal stiffness of nodules, which might be due to the fact that this sample only contained TIRADS 4 of TNs, which had a higher proportion of calcification.

If the *E*_shell_/*E* ratio was higher than 1, the risk of malignancy of TNs was higher; conversely, the possibility of being benign was greater. This study also showed that the *E*_shell_/*E* ratio of malignant TNs was higher than that of benign TNs, and the difference was statistically significant (*P* < 0.01). The advantage of *E*_shell_/*E* over *E*_shell_ or *E* was that it avoided the differences between observers.

The objective of the traditional statistical analysis is to establish an accurate and simple model for the relationship between independent and dependent variables as much as possible. When the relationship between independent and dependent variables is relatively simple, the efficiency of this statistical analysis is higher. However, when the relationship is complex, such as a nonlinear functional relationship, the traditional statistical analysis becomes difficult, its efficiency is low, and the requirements for the analysis are high. According to the purpose of analysis, a decision tree divides the total study population into several relatively homogeneous subpopulations based on certain characteristics, which can handle the complex relationship between independent and dependent variables in a relatively simple and easy way. CHAID is the most basic and simple classification algorithm. Some studies applied the classification algorithm to the diagnosis of breast nodules and proved that similar algorithms could improve the diagnostic accuracy, no matter for breast magnetic resonance or breast ultrasound (20, 21). Kapetas et al. showed that the application of CHAID achieved the highest diagnostic accuracy and the sensitivity of 98.46%. This study also achieved similar results in the diagnosis of thyroid ultrasound. The classification algorithm selected *E*_shell_ and *E*_shell_/*E* among the three quantitative parameters. Compared with individual quantitative parameters, the AUC of the classification algorithm was the highest, with a sensitivity of 98.7% (223/226). Such a high sensitivity ensured that the omission of malignant TNs could be avoided as much as possible in clinical work, which was a prerequisite for us to reduce unnecessary FNA. In Node 4, 118 of 121 TNs were correctly diagnosed, implying that 118 unnecessary FNA were avoided in this sample using this algorithm. At the same time, the probability of malignancy was only 2.5%, which was even lower than the upper limit of malignancy for TIRADS 3 TNs (5%). Therefore, short-term follow-up might be appropriate for these TNs, with a lower risk of missing a large number of thyroid cancers. In Nodes 2, 3, 5−10, the probability of malignancy was more than 23.9%, and therefore the FNA was recommended in all cases.

Therefore, in the face of the clinical management of a large number of TIRADS 4 TNs, the application of a classification algorithm to establish a simple model could quickly and accurately find potentially benign TNs and suggest their follow-up, which helped achieve our goal of reducing FNA.

This study had some limitations. First, as a provincial hospital, many patients were initially diagnosed with thyroid cancer in subordinate hospitals and suggested to come to our hospital for further examination. In addition, some patients with no certain pathological diagnosis (include Bethesda III and IV) were excluded from this study, resulting in some sampling bias. Second, some TNs were discarded because they could not be used to measure the stiffness of the surrounding 2-mm tissue. Finally, this was a single-center study with a relatively small sample size. Further testing of this classification algorithm requires multicenter studies with a large sample size.

## Conclusion

In conclusion, a simple classification algorithm was applied to improve the diagnostic performance of ultrasound elastic parameters of TNs, avoiding FNA in 57.3% of nodules.

## Data Availability Statement

The raw data supporting the conclusions of this article will be made available by the authors, without undue reservation.

## Ethics Statement

The ethics committee of the First Affiliated Hospital of the University of Science and Technology of China (USTC) approved this prospective study. Written informed consent to participate in this study was provided by the participants’ legal guardian/next of kin.

## Author Contributions

LX, XL, XY, YC, NH, and LH participated in literature search, data acquisition, data analysis, or data interpretation. XL and LH conceived and designed the study, and critically revised the manuscript, performed the research, wrote the first draft, collected and analyzed the data. XL, NH, and LH participated in paper writing and revised the manuscript. All authors contributed to the article and approved the submitted version.

## Conflict of Interest

The authors declare that the research was conducted in the absence of any commercial or financial relationships that could be construed as a potential conflict of interest.

## Publisher’s Note

All claims expressed in this article are solely those of the authors and do not necessarily represent those of their affiliated organizations, or those of the publisher, the editors and the reviewers. Any product that may be evaluated in this article, or claim that may be made by its manufacturer, is not guaranteed or endorsed by the publisher.
